# *Fast* Refugee Protection: Temporality and Migration Control

**DOI:** 10.1177/09646639211015340

**Published:** 2021-05-10

**Authors:** Azar Masoumi

**Affiliations:** Carleton University, Canada

**Keywords:** Space, state-controlled migration regulation, technologies of migration control, temporality

## Abstract

This article explores the temporality of migration control through an analysis of refugee claim processing in Canada. I draw on organizational reports, commissioned studies, media reports, interviews and archival data to argue that time is a key technology of state-controlled migration regulation. I show that temporal technologies have long been used to both control the access of migrants and the labour of civil servants. Furthermore, I show that procedural temporalities have been consistently manipulated to reflect and facilitate growing restrictionism in Canadian migration regulation. In short, I suggest that migration regulation regimes devise and use temporal technologies to block, deter or delay access to rights to unwanted and unauthorized migrants, and to reduce the cost of doing so where possible.

There are hundreds of thousands of people who patiently wait to come to Canada through legal means, and it’s just not fair. It’s not right for people to jump on a plane, come here to make a refugee application, even if they don’t meet the definition of refugee.

Minister Jason Kenney (CBC News, 2009)

## Introduction

Migration regulation is, of course, a spatial political project: it is one that aims to regulate, control, and restrict access to national *territories* and the civil and citizenship rights that are associated with them. But territories do not exist outside of time and, hence, their spatiality is always already temporal(ized). Take, for example, the Designated Countries of Origin (DCO) policy, introduced in 2012 by the then Conservative government of Canada (2006–2016). The DCO was part of a repertoire of restrictive policies implemented in (and at times imposed on) the Canadian refugee protection regime to, purportedly, ‘deter abuse of the refugee system’ (Government of Canada, 2019). The logic of the policy was simple and quite clearly spatialized: the DCO provided a list of *‘places* in the world where it is less likely for a person to be persecuted compared to other areas’ (Government of Canada, 2019, emphasis mine). Places that, in the estimation of the Canadian government and by the Immigration Minister’s decree, were ‘generally considered safe’ and free from abuses of human rights. Refugee claims from these *places*, which were supposedly plentiful, were believed to be largely unfounded (and a drag on limited time and resources). Thus, these claims were scheduled to be ‘processed *faster*’ (emphasis mine), a temporal manipulation of normal processing times that was meant to ‘make sure that people in need get protection *fast*, while those with unfounded claims are sent home *quickly*’ (Government of Canada, 2019, emphasis mine).

The DCO soon became highly controversial because, among other things, it included countries that, as it happened, could not be considered ‘safe’ by impartial third-party observers. For example, both Hungary and Mexico were placed on the list of ‘safe places’ while neither, according to well-documented accounts produced by international human rights organizations, could be considered safe for certain minorities (such as Roma in the case of Hungary^1^) or due to rampant state and extra-state violence (such as that transpiring during drug wars in Mexico^2^). A few unresolved legal challenges^3^ and several hundred deportations later, the DCO was eventually laid to rest in the archives of Canadian migration regulation history in 2019, when the Liberal government removed all countries from the list and de facto annulled the policy (Government of Canada, 2019).

In addition to representing a troubling moment in the evolution of restrictionist migration control, the DCO is a clear example of the intertwined spatial and temporal technologies of migration regulation. In the regulatory logic of the DCO, spatial considerations (i.e. people from which *places* are likely to gain/not gain access to *here*) are at once enmeshed with temporal ones (i.e. people from which places are to be processed at which *pace*): the access of people from ‘safe’ *places* (spatial) to *here/Canada* (spatial) is, in part, regulated and restricted by processing their refugee claims *faster* (temporal) and sending them *home* (spatial) *quickly* (temporal). Spatial regulation of migrants is at once temporal(ized).^4^

This paper explores the temporality of state-controlled migration regulation through an analysis of in-land refugee claim processing in Canada. In the Canadian system, claims made from inside national territories (i.e. in-land) are processed by different bodies and hence are subject to different sets of protocols compared to those made abroad, for example at Canadian visa offices or through international agencies. In the context of this discussion, I use the term *temporal technologies* to refer to technologies that work to control the temporal dimensions (such as time-use and timing) of state procedures. I show that temporal technologies have long been used to reinforce both the spatial control of migrants and the managerial control of civil servants. Furthermore, I show that temporal technologies have evolved over time to facilitate growing restrictionism in migration regulation. All in all, I present state-controlled migration as a project of temporal regulation and manipulation; I argue that migration regulation regimes devise and use temporal technologies to block, deter or delay access to unwanted and unauthorized migrants, and to reduce the cost of doing so where possible.

I draw on organizational reports, commissioned studies, interviews and archival data to identify and explore the temporal technologies that have been utilized in administering Canadian refugee protection. The body of data that informs this paper was collected as part of a larger study on bureaucratic administration of refugee protection in Canada. The organizational reports and commissioned studies consulted here are publicly accessible and were located through libraries and government websites. Archival documents were acquired from the Library and Archives Canada and accessed following Access to Information and Privacy Request (ATIP) reviews. Semi-structured interviews were conducted with former refugee status adjudicators and refugee lawyers in 2016 and 2017. Participants were recruited through connections with refugee law offices and advocacy organizations as well as snowball sampling. The combination of these diverse methods of data collection allowed a view of Canadian refugee protection at the individual, bureaucratic, political and historical levels. Data from various sources were analysed with and through one another and with an eye for producing a cohesive account of the evolving temporal technologies of refugee claim processing.

The next section of this paper theorizes the relationship between time, space and state-controlled migration regulation to understand the significance of temporality in regimes of migration control. In the remaining sections, I turn to analysing temporal technologies, their logics, evolution and intended and unintended consequences in the Canadian regime. First, I examine ‘general’ temporal technologies of refugee claim processing in Canada; these include technologies that are implemented in relation to *all* claims. This discussion will demonstrate the dual utility of these technologies in maximizing the extractable value of the labour of those who process refugee claims, and minimizing the access of those who wish to receive protection. Second, I explore ‘targeted’ temporal strategies; these include technologies that are implemented in relation to only a portion of claims. I show that targeted technologies have contributed to the administration of an increasingly restrictive, and uneven, regime of refugee protection. I conclude the paper by presenting temporal technologies as impactful yet imperfect devices of restrictionist migration control; the imperfections, or ‘cracks’, of temporal technologies, I suggest, produce unintended and at times surprisingly expansive outcomes.

## Time, Space and Migration Regulation

At first glance, any assertation about the entanglement of space and time may seem so obvious as not to warrant note. Of course, spaces exist within time. *All* spaces are temporalized. Yet, there is something specific, and specifically political, about temporalization of space. Fabian (1983), for instance, has shown how temporalization of colonies as regressive spaces, as spaces stuck in earlier eras of history, evolution, and civilization, has been critical to envisioning and mobilizing colonial projects. In other words, spaces are temporalized in specific ways by specific forces for specific reasons. As Grabham (2018) contends, by virtue of being constituted by assemblages of human actions and matters, (legal) temporalities necessarily engage questions of power. Thus, the temporalization of space, and indeed temporalization itself, is political.

Hanchard’s (1999) insightful conceptualization of ‘racial time’ captures the political underpinnings of temporality. In Hanchard’s conceptualization, racial time refers to ‘the inequalities of temporality that result from power relations between racially dominant and subordinate groups’ and is reflected in ‘unequal temporal access to institutions, goods, services, resources, power, and knowledge’ (p. 253). Hanchard suggests that under politically uneven regimes (such as those of neo/colonialism) time is manipulated differently for different purposes and different populations. This ‘temporal discrepancy’, Hanchard notes, means that time, for instance, ‘move[s] more quickly for the extraction of capital, resources, and surplus value by the colonizers but less so for the educational development of Ghanaian children and the training of their teachers, or for the use of some of those extracted resources for positive national development’ (p. 263). Racial time distributes speed and stagnation unequally, so that some, for example African Americans, receive resources only *after* whites, a situation that has black Americans ‘wait for nearly everything’ (p. 263). In effect, as Mills (2014) subsequently notes, temporal technologies of oppressive regimes redistribute time by ‘taking time *away* from [some] people’ (p. 27) and transferring it, and its values, to others. In other words, time is both a site and a mechanism of power.

Despite the political significance of temporality, temporal dimensions of migration regulation have been largely overlooked in favour of questions of space and spatial control. The scholarship on migration regulation has consistently advanced epistemologies that attend to the territorial anxieties (from a state-centric, nationalist perspective) or critiques (from a critical, progressive standpoint) of nation-states. As a result, we have overwhelmingly prioritized studying space over time, despite the fact that neither exists outside the other.

Of course, the attention to the spatial dimensions of migration regulation has been invaluable. The critical scholarship produced by political scientists, sociologists and human and political geographer have provided insightful analyses of spatial technologies of migration regulation. Thanks to this scholarship, we know much about how states deploy space, such as borders (Pratt 2005, 2010; Pratt and Thompson, 2008; Salter and Piché, 2011; van der Woude and van der Leun, 2017; Walters, 2006; Zaiotti 2011), detention centres (Loyd and Mountz, 2018; Nethery and Silverman, 2015; Wilsher, 2012), islands (Lemaire, 2014; Loyd and Mountz, 2014; Mountz, 2011, 2015), international waters (Brouwer and Kumain, 2003; Kim, 2017; Markard, 2016; Sönmez, 2016), airports (Alpes, 2015; Sanchez et al., 2016; Sulmona et al., 2014) and even the space onboard ships (Ellebrecht, 2020; Klein, 2014; Koka and Veshi, 2019), aircraft (Walters, 2015), and buses (Teunissen, 2020) for the purposes of restrictive migration control. We know, for instance, that states routinely take advantage of the ambiguous legal standing of certain spaces, or otherwise manipulate the standing of others, to produce zones of exception (Agamben, 2005) beyond the ordinary bounds of rights. We know that these legal-spatial strategies effectively impede unauthorized access to rights that would have been otherwise conferred on migrants (for instance see Mountz, 2010). In other words, we know that much of migration regulation is carefully arranged and operationalized around the nexuses between space and the law.

As insightful as these studies have been, they have neglected explicit analysis of the *temporal* dimensions of spatial technologies. This is despite the fact that temporality is, in fact, integral to the efficacy of spatial technologies of migration control. For example, intercepting unauthorized travellers onboard of boats or holding migrants in ad-hoc zones of transit on islands, serve the purposes of migration restriction only when accomplished *before* migrants assume the chance to step foot on the right-conferring soil of national territories. In other words, spatial technologies of migration regulation rely on delicate temporal considerations and specificities to be of any use to states.

But temporal technologies of migration control are not, nor should they be assumed to be, only subsidiary to spatial technologies. Temporal technologies are significant devices of governance in their own right. As Marx (1967 [1867]) showed convincingly in Volume I of Capital, regulating time is central to the workings of power (and accumulation of capital). In reading Marx, Tombazos (2013) writes that, ‘[c]apital, like any other economy, is a specific organisation of *time* obeying its own immanent criteria’ (p. 3, emphasis mine). Time is a key juncture through which subjugation is materialized and governance operationalized. Indeed, time and temporal control is a central feature of power; the powerful, for example, are waited upon while the powerless wait. Put differently, waiting is a political temporal *imposition* (Schutz and Luckman, 1974). As such, those who work to the pre-planned pace of the assembly line are situated gravely differently (socially, economically and politically) compared to those who plan such timed rhythms, benefit from their implementation, or do both. Much of social and political control is achieved through accumulating, if not monopolizing, mastery over time, be it productive, reproductive, or procedural.

In the realm of migration control, temporality has recently received some attention. Khosravi (2018, 2019), for instance, has explored some of the temporal dimensions of migration control and unauthorized travel. For instance, he has considered the temporality of migration control both in terms of the time lost in waiting, delays and recirculation of migrants through deportation, as well as the redistribution of the economic value of this lost time from unauthorized residents to nation-states. These explorations foreground key questions about the temporality and temporal consequences of migration control.

To be sure, specific temporal dimensions of migration regulation have long been studied. For instance, anthropologists, ethnographers and social workers have for decades considered the experiences and consequences of waiting (Akram et al., 2014; Eyre, 1990; Cabot, 2014; Greene, 2019; Hvidtfeldt et al., 2018). Thanks to this scholarship, we have an understanding of how delays, queues and wait times are experienced and survived by migrants and refugees. Through these studies we know that the time lost in waiting for resettlement, legal status or repatriation costs migrants in terms of their physical, psychological and economic health and their aspirations for keeping to any semblance of a normative life-course. We know that the unequal distribution of wait times by regimes of migration control amount to, to borrow from Hanchard (1999), ‘negation of productive life’ (p. 265). In short, these studies capably highlight the *temporal* subjugation of migrants to migration regimes; they suggest that migrants’ subjugation is temporal as well, and as much, as spatial.

Despite their insightfulness, these studies offer little in the way of understanding the temporality of operations of power, as opposed to the temporal experiences of subjugation. For instance, we never learn what it is that wait times achieve for migration regimes, or how they are determined and imposed. In short, we learn little about temporality as a *technology* of state-centred migration control. This approach treats temporality as an *experience* (of the migrant), rather than a *device* (of the state), skewing attention from those who impose to those who receive impositions. Yet, as Cohen (2018) notes, time ‘is a [frequently deployed] tool in the arsenal of a state’ (p. 32). How does time, then, feature in migration regulation regimes beyond migrants’ phenomenological experiences of delays, waiting and temporal subjugation? How is time actively used and manipulated by migration control regimes to restrict access to mobility, residency, and citizenship? In what ways do migration regimes rely on and utilize time as a tool for keeping undesired migrants out or turning them away?

Temporal technologies of migration regulation are particularly interesting in light of their distinctions from spatial technologies, and in terms of their highly uneven usability to states versus migrants. For while technologies of migration regulation are heavily spatialized (for example by being articulated around borderlines^5^), so are migrants’ methods of unauthorized travel: unauthorized migrants, and those who aid their travels, continually develop innovative spatial strategies of their own to circumvent restrictions and reach the more rightful sides of migration regimes. In fact, unauthorized travel in and of itself may be understood as migrants’ strategic spatial manipulation of restrictive migration systems: unauthorized migrants literally place their bodies in spaces where they are not intended or allowed. Of course, migrants’ spatial strategies can be highly involved and creative. For example, migrants may, as large numbers of Haitians did to reach Canada in 2017 and 2018, take advantage of spatialized legal loopholes to gain access to refugee systems through ‘irregular’ border crossings as opposed to submitting themselves to the restrictive and unwelcoming protocols of official ports of entry (Samuel, 2018).^6^

Migrants also attempt to temporally manipulate migration regulation regimes. They may, for example, embark on unauthorized border-crossing under the shadow of the night or time their trips so that (if things go as planned) they arrive at their destinations at certain times. But beyond these ‘soft’ attempts at manipulating time, migrants have little control over the temporal dimensions of their spatial maneuverings. States, on the other hand, are far better positioned to use time for the purposes of migration regulation and against migrants. In fact, as I hope to show, states are particularly likely to resort to temporal technologies when confronted with migrants’ successful spatial manipulation of migration restrictions: while much spatial strategizing goes into keeping unauthorized migrants *far* and *away*, a whole host of temporal technologies are reserved for and applied to those who somehow manage to make it *here* (such as *in-land* refugee claimants). In effect, when migrants successfully manipulate migration restrictions spatially, states manipulate their access temporally (for example by determining how quickly their refugee claims will be processed). These temporal manipulations, of course, constitute a shift from the temporal logics of colonial control, in which the space of the Other is breached (economically, politically, militarily, and otherwise) to purportedly traverse the colonized forward in time: spatial imposition was justified via temporal ‘advancement’. In contemporary logics of migration control, the configurations of spatial and temporal control are reworked such that now the Other assumes mobility into ‘our’ space and is consequently contained in a time quasi-legally set to before their full arrival (by way of, for instance, detention on islands, transit zones and airports) as to limit their access to rights. Notwithstanding these distinctions, both systems manipulate time to regulate access to space. Hence, a fuller view of political control, including over migration, requires considering how spatial access is regulated via logics and technologies of time.

## Time Is Money: Labour, Restriction and Temporality

Time has long been a focus of administrative tinkering in the Canadian regime of refugee protection. Ever since the current system was established, administrators have worked hard to optimize the use of time, and maximize its extractable value, in claim processing procedures. The focus on time has at points bordered on administrative obsession, as reflected, for instance, in close tracking of all the temporal dimensions of refugee claim processing through daily, weekly, monthly, quarterly and yearly reports.^7^

The obsession with time is perhaps not surprising given the context in which the Canadian regime began its operations. The quasi-judicial tribunal that shoulders the work of in-land refugee claim processing in Canada was created in 1989, a time of rapid neoliberal restructuring in much of the global north (Harvey, 2005). The largely humanitarian mandate of refugee protection, which Canada took on by becoming a signatory of the Convention Relating to the Status of Refugees in 1969 (See UNHCR, 2019), was somewhat a historic mismatch with the increasingly economized demands of neoliberal governance.^8^ In this context, it was paramount that the international obligation to refugee protection was carried out as cost-consciously (i.e. cheaply) as possible. Cost-consciousness, in turn, required time-consciousness. As market logics had long known, time is costly; hence, diminishing cost requires controlling time, a logic plainly evident, for example, in the temporally standardized model of Fordist production.

In the Canadian regime, tampering with procedural temporality largely focused on the mutually entangled goals of shortening processing times and increasing their output. Unexpected and drastic increases in caseloads, for instance the doubling of the number of incoming claims in 1989–1990 (IRB, 1990, 1991), only strengthened the incentive for efficiency. Over the years, claim processing procedures were manipulated several times to maximize the ratio of output to time. Even the design of the determination process was altered to condense time-use. For instance, in 1993, the then two-staged adjudication procedure was reduced to a single stage (IRB, 1994).

Of course, regulating the conditions and conduct of labour was key to optimizing time-use and its associated costs. Subsequently, the pressures of neoliberal efficiency were swiftly downloaded on the street-level bureaucrats (Lipsky, 1980) who shouldered the daily work of refugee claim processing. Canadian administrators used a variety of structural and case-level methods to maximize the amount of extractable labour from employees, particularly those who adjudicate the status of cases. In some instances, significant features of the determination process were altered to optimize the output of adjudicators’ time. For example, refugee claims were originally legislated to be heard by two decision makers; only one decision maker needed to approve the claim for refugee status to be granted. This model offered a modest level of protection against unilateral negative decisions. However, it severely limited the number of claims that could be finalized by existing complements of decision makers. Hence, the Canadian system was gradually transitioned to single-person adjudication panels.^9^

Single-member panels contributed greatly to increasing case processing outputs. However, the increase in output was not without cost to adjudicators. Deciding cases without collegial input demanded substantially more from individual decision makers. The weight of this change was particularly felt by new and inexperienced adjudicators who could no longer benefit from the organic forms of mentorship that two-person panels provided (Interview with former decision maker, 6 October 2016). In other words, increasing the output of adjudications required extracting more labour from individual adjudicators’ time.

Adjudicators were also, and often, subjected to various labour management techniques that aimed to increase the output of their time. Some of these techniques were designed simply to press more out of adjudicators. For example, the volume of output expected of decision makers grew substantially over the course of the 1990s, from an average of 2.5 cases per week in the early years, to four weekly cases by the end of the decade (Interview with former decision maker, 6 October 2016). Further, the length of time adjudicators spent in hearing rooms with claimants was trimmed where possible. For example, in 2001–2002, a complex case management system was introduced to divert claims to four streams based on their levels of complexity: ‘manifestly well-founded’ claims were sent to an expedited interview (in lieu of a formal hearing), ‘straightforward’ cases were directed to short hearings, cases of average complexity were decided in regular hearings, and only particularly complex claims were given a long hearing (IRB, 2002). The time freed through these practices could now be dedicated to processing more claims.

In addition, various techniques were implemented to induce optimal extraction of labour. These included infrastructures of individual and collective (self-)surveillance. For instance, performance reports on individual decision makers and entire offices were regularly produced and presented in meetings to encourage speed of claim processing (Interview with former decision maker, 6 October 2016). Moreover, decision makers were steered away from circumstances that would lead to ‘time waste’; for example, decision makers’ discretionary latitude in adjourning and postponing hearings were discouraged (Interview with former decision maker, 6 October 2016), as adjournments and postponements had long been considered a major source of wasted time and scheduling energies (see for instance IRB, 1991). Of course, given decision makers’ well-protected political and adjudicative independence in the Canadian system,^10^ these forms of management were conducted largely indirectly, even if not wholly discreetly.

Importantly, temporal technologies of claim processing combined subjugation of workers with that of refugee claimants. For example, in order to force speed and filter out ‘fraudulent’ claimants, the Balanced Refugee Reform Act (2010) and the Protecting Canada’s Immigration System Act (2012) imposed extremely short timelines for submitting *and* processing refugee claims.^11^ The new timelines disadvantaged claimants by not allowing them sufficient time to find lawyers, gather documents, and prepare well-supported cases (Interview with refugee lawyers, September 6, 2016 and February 20, 2017). Meanwhile, the imposed timelines disturbed the normal rhythms of case processing and demanded faster turnovers than existing resources permitted (IRB, 2014). In short, forced speed was to the detriment of both claimants (whose access was further restricted) and civil servants (whose labour was further extracted). In effect, the regulatory logics that meant to rid the system of ‘fraudulent’ claimants were equally invested in ridding it of ‘inefficient’ work/er; temporal technologies of migration control fused restriction of access with extraction of labour.

## Time Is Deterrent: Temporalities of Restrictionism

Tinkering with procedural temporality to reduce operational costs is perhaps a general feature of neoliberal states. However, temporal manipulation is also a specific technology of restrictive migration control: migration regulation regimes manipulate time not only to cut costs, but to impede unwanted migrants. Of course, temporal technologies are *temporal*; they change with time and in response to evolving political and administrative agendas of migration control. In Canada, two diverging temporal logics have dominated the design of claim processing procedures in various periods, marking a gradual shift from a more permissive regime of refugee protection to an increasingly more restrictive one in more recent years.

In the early years of the Canadian system, the temporality of refugee claim processing was considered largely in its implications for refugees’ access to protection. Even before the current system was established, fast refugee protection was associated with meaningful responsiveness to refugees. For example, in his commissioned report on the Canadian refugee status determination system in 1985, Rabbi Gunther Plaut argued that good refugee protection required ‘quick readiness to acknowledge [refugee needs]’ (p. 6). Importantly, temporal swiftness was not valued simply for its contribution to cutting unnecessary costs, but as a moral imperative in itself: ‘[t]here is a human dimension to saying a ready ‘yes’ to a refugee’ (p. 6). In other words, ‘good’ refugee protection was to be fast. Temporality was a matter of responsiveness.

The logic of temporal responsiveness was influential in shaping early temporal technologies of refugee claim processing in Canada. The earliest procedures devised to increase the speed of claim processing, the expedited and the simplified inquiry process (inaugurated in 1990), clearly followed this logic. These two procedures fast-tracked ‘evidently well-founded’ claims, routinely determined by their origins from countries that presented well-documented records of human rights violations. In the early 1990s, these included El Salvador, Iran, Lebanon, Somalia, and Sri Lanka (IRB, 1991).^12^ Claimants from expedited countries normally had extremely high acceptance rates, ranging from 80% to more than 90% (see for example IRB, 1990, 1991). Because of their perceived unambiguous nature, these claims were believed to be able of being adjudicated quickly and with fewer resources. For example, the simplified and expedited processes allowed claims to be adjudicated in short informal meetings rather than full two-adjudicator hearings.^13^ In effect, these early fast-tracking systems aimed to quickly conclude the adjudication of claims that were likely to receive a positive decision in normal proceedings; shortened processing times were meant to quicken ‘real’ refugees’ access to protection. In other words, these early temporal technologies were designed with an in-built bias towards fast *acceptance*.

Yet, temporal responsiveness was at once balanced by a far less permissive logic. For as long as swiftness has been considered integral to proper refugee protection, it has also been understood as an effective strategy for deterring those who supposedly wish to jump immigration queues by making a ‘fraudulent’ claim (see Plaut, 1985). According to the temporal logic of deterrence, fast claim processing eliminates (some of) the incentive for ‘abuse’ by shortening the length of time that filing a refugee claim buys claimants in Canada.

The logics of temporal responsiveness and deterrence have long been entangled. Indeed, deterrence was thought to facilitate responsiveness by eliminating unnecessary caseloads, while responsiveness was meant to naturally deter ‘fraudulent’ claims (see for example IRB, 1990, 1991, 1992). However, over the years, deterrence became an increasingly pronounced logic in the administrative visions of refugee claim processing. Within a few years, the logic of deterrence created a temporal ripple effect across the Canadian migration control apparatus. For example, the claim processing bureaucracy began to call for timely removal and deportation of rejected claimants (IRB, 1991, 1992), a mandate far beyond the largely humanitarian scope of its own work. As it appeared, the temporal logic of deterrence was only effective if properly synched with the temporality of deportations; all protocols needed to be quickened for claimants to receive the message of deterrence intended by fast processing of claims.

Over the years, the shift from temporal responsiveness to deterrence became progressively more complex and systematized. In the mid-1990s, administrators began to manipulate the scheduling order of adjudications to prioritize the processing of supposedly ‘unfounded’ claimants; in a reversal of earlier practices, expedition began to be used as a basic temporal technology for producing fast *rejections*. By the late 1990s, temporal technologies of deterrence became considerably more involved. In 1998, the Canadian regime began to produce and endorse a ‘model’ of negative decisions, known as Lead Cases, that were expected to be consulted, if not replicated, by decision makers in deciding Roma claims from Hungary. In effect, Lead Cases normalized and incentivized systematic rejection of claims by making negative decisions far less time and labour-intensive. Significantly, unlike the earlier expedited processes, the temporal technologies of the late 1990s were designed with an in-built bias *against* accepting claimants as refugees. This shift in conceptions of procedural temporality was highly impactful: the new technologies helped reject targeted claimants quickly and in great numbers. For instance, within one year of the implementation of Lead Cases, acceptance rates of Hungarian Roma claimants dropped from 71% to 16%. Following their repeal in the Federal Court of Appeal in 2006,^14^ the rates improved substantially to 62% (Levine-Rasky, 2016: 115). In the interim period, thousands of Roma Hungarians were turned away from protection.

Temporal technologies of deterrence were further systematized in the ensuing decades. Under the direction of the Conservative immigration Minister Jason Kenney (2008–2013), fast-tracking came to assume a clear tone of deterrence, far removed from its original emphasis on responsiveness. Most notably, the Minister introduced the Designated Countries of Origin (DCO) policy in 2012, a system of fast-tracking that was externally control by the political authority of the Minister himself.

The DCO was openly advertised as a system of deterrence (see Government of Canada, 2019). In sharp contrast with the earlier expedited processes, the DCO was designed to fast-track supposedly ‘unfounded’ claims from ‘safe’ countries. The purpose of fast-tracking was to expedite negative decisions and send ‘fraudulent’ claimants home quickly. While claims were normally scheduled for a hearing within 60 days of being filed, DCO claims were heard in only 30 to 45 days. The truncated scheduling period gave DCO claimants little time to prepare strong cases. Further, DCO claims were excluded from the right to appeal negative decisions through the claim processing tribunal. To make matters worse, quick removal of rejected claimants from Canada practically left no time for an appeal through the slow-moving court system, making negative decisions quick and practically irreversible (Levine-Rasky et al., 2014).

In sum, quickening the pace of claim processing was not simply a neutral administrative tool for improving efficiency and productivity. Rather, expedition was used strategically to manipulate migrants’ access to legal status. As Canadian administrators and political authorities quickly discovered, *fast* refugee claim processing readily accommodated restrictionism. Indeed, over the span of only a decade and a half, temporal technologies of claim processing in Canada moved away from a logic of humanitarian responsiveness to prioritize deterrence and restriction. This shift, of course, was consequential for those who sought refuge in Canada: being fast-tracked to receive refugee protection is radically different from being expedited towards a rejection. With expedited rejections established as the primary goal of fast refugee protection, procedural temporality became an effective tool for migration restrictionism.

As effective as temporal technologies have been in regulating access to refugee status, they have not always produced the desired outcomes. In fact, temporal technologies of migration regulation at times produce significant unintended side-effects. A case in point is the aforementioned imposition of impractical refugee claim processing timelines through the Protecting Canada’s Immigration System Act (2012). While these timelines were unmistakably intended to restrict access to protection, they inadvertently improved some claimants’ chances of being accepted as refugees. This was particularly true for those fleeing rapidly changing national contexts of persecution: fast resolution of claims meant that claimants could no longer be rejected on the grounds of perceived improvements in the conditions of persecutory home countries (Interview with refugee lawyer, September 6, 2016). In other words, temporal technologies of restrictionism presented unexpected openings for some groups of claimants; these temporal ‘cracks’ undid some of the intended goals of restrictionist migration control.

The imposed timelines created even grander unintended consequences, in the form of the ongoing debacle with the so-called ‘legacy cases’: the over 30,000 cases that were filed before the new Act came into force on December 15, 2012, and hence fell out of its purview. The new temporal stipulations, as it appeared, had not seriously considered these ‘legacy’ claims nor did they provide any method, time or budget for their resolution (IRB, 2014). Thus, legacy claims were perpetually pushed to the back of the queue to make processing new claims within the legislated timelines possible. As a result, their resolution has been years in the making: today, 8 years after the Act, 50 legacy claims remain to be resolved (IRB, 2020). In effect, expedition of the processing of new cases was achieved at the expense of excruciating slowing of the processing of older claims, many of which had already been in the system for a few years even before the Act came into force. The result has been a system of polarized temporalities: quick resolution of new claims that barely allows enough time for proper case-preparation,^15^ and years of delay in processing legacy claims that cost migrants in high tolls of deteriorating mental health, prolonged interruption in family life, and extended economic and social limbo (see Keung, 2016, for one example). The attempt at fastening procedures has ironically created substantial slowing; and yet, both the fast and the slow have come at high costs to those seeking refuge in Canada.

## Conclusion: Time, Control and ‘Cracks’

This article explored temporal technologies of migration control in the Canadian regime of refugee claim processing. I showed that procedural temporalities have long been manipulated for the dual goals of improving the productivity of labour (and hence controlling costs) and restricting the access of unwanted migrants (and hence controlling migration). I suggested that temporal technologies of refugee claim processing shifted from an emphasis on responsiveness to deterrence: fast-tracking moved away from the logic of quickly accepting ‘likely’ refugees to quick rejection of supposedly ‘unlikely’ ones. Hence, procedural times were shortened not solely to improve rates of output, but to also improve restrictive control. In short, temporal technologies reflected and facilitated growing restrictionism in Canadian migration control.

Of course, restrictionist temporal technologies of migration control are by no means distinctive to Canada. In fact, many of the temporal technologies discussed here are common features of migration control regimes across the global north. For instance, the punishing temporal logics of the Canadian DCO are paralleled by those of the infamous and now repealed Detained Fast Track (DFT) and the Non-Suspensive Appeal (NSA) procedures in the UK. While the DFT used spatial control (through detention) to accelerate negative decisions, the NSA fast-tracks the rejection of supposedly ‘clearly unfounded’ claims, often (but not exclusively) determined based on designations of ‘safe’ countries. Similarly, the misleadingly titled Migrant Protection Protocols (MPP) (more aptly known as ‘Remain in Mexico’) uses fast-tracking against claimants arriving in the US through third countries, effectively blocking the access of Central Americans who, by necessities of geography, travel via Mexico (i.e. a third country) in search of American protection. Similar accelerated asylum procedures also exist in Germany, France and Italy,^16^ to name only a few countries in western Europe, despite the fact that procedural speed is associated with attenuation of legal process and deterioration of access to rights (Hambly and Gill, 2020). In short, states across the wealthy liberal world manipulate procedural temporalities in order to exert restrictive control over migration. The global relevance of these temporal technologies is perhaps not surprising, given that states often design their migration control systems in consideration and consultation with those of other states. Indeed, with border control increasingly treated as a collaborative project (Doyle, 2010; Lawson and Bersin, 2020), temporal technologies of migration control are bound to be shared, replicated and reproduced across political jurisdictions.

This article presented temporal technologies as highly effective devices in mediating access to protection and rights. The global prevalence of these technologies highlights their centrality to border and migration control. Indeed, in and beyond regulation of migration, time is both a critical site and a mechanism of power. Temporal manipulations, as I have suggested, reflect and (re)produce polarized distributions of power. Whether formulated in acceleration (such as in refugee fast-tracking systems) or delay (for instance in services to colonized and racialized populations), temporal technologies actively produce (differential) political effects. In fact, spatial political control is, in part, temporally regularized: access to space and mobility is critically regulated through control over time.

Despite the significance of temporal technologies of migration control, it is important to note that these technologies, like any other technology, never fully lend themselves to the will of state agents. As my discussion of the unintended consequences of the Protecting Canada’s Immigration System Act (2012) suggests, unexpected outcomes can and do follow the temporal tinkering of migration regimes: restrictionist acceleration programmes at times unintentionally improve access to protection, while delays may provide opportunities for unauthorized migrants to develop grounds for status. Time is a finicky site for the exertion of state control. While often greatly costly to migrants, the unintended outcomes of these technologies reveal temporal ‘cracks’ in otherwise seemingly seamless regimes of migration regulation.

## Notes

1. See Human Rights Watch (1996, 2015), Gall (2013), Minority Rights Group International (2018).

2. See Pichico (2013), Amnesty International (2015), Wilkinson (2018), Human Rights Watch (2019).

3. The DCO was successfully challenged in the Federal Court on the basis of non-compliance with the Canadian Charter of Rights and Freedoms (CCR, 2015). In response, the Conservative government initiated an appeal of the ruling. When Conservatives conceded to Liberals in the 2015 election, the appeal was publicly abandoned (CBC, 2016).

4. The temporality of migration control is also evident in immigration detention: in addition to spatial confinement, detention confiscates migrants’ time in a process unmistakably resembling a, albeit undefined, prison sentence.

5. Of course, if we take racialized bodies as embodiments of borders, as Khosravi (2010) suggest we should, borders become far beyond spatially static geographical points on the edge of national territories. ID checks of racialized folks suspected of being ‘undocumented’, for example, suggest that borders are marked on the migrant body in all places and at all times.

6. In 2002, Canada and the US singed the Safe Third Country Agreement (in effect since December 29, 2004), to prevent access to each country’s refugee system to those who enter from the other country (for more see Government of Canada, 2020). The Agreement is based on the assumption that both countries are ‘safe’ for refugees, and hence refugees should make their claim in the first country in which they arrive. The Agreement has been highly effective in curbing access to Canada’s refugee system (Hyndman and Mountz, 2007); however, the provisions of the agreement do not apply to ‘irregular’ border crossings at points other than those designated as official ports of entry.

7. See, for example, the reports included in Refugee and Displaced Persons- General-Immigration and Refugee Board, Department of Employment and Immigration fonds, Library and Archives Canada, Ottawa, Canada.

8. For more on the economization of political life under neoliberalism see Brown (2015).

9. Administrators began diverting only a portion of claims, with claimants’ consent, to single-person panels in late 1989 (IRB, 1990). The diverted claims were generally considered to be ‘well-founded’ and tended to be accepted at high rates (see for instance IRB, 1991). The number of diverted claims were gradually increased in the ensuing years. By 1992, 35% to 40% of total cases were adjudicated outside of the legislated two-person hearing process (IRB, 1993). The adjudication process was eventually transitioned fully to single-person panels in 2001 by the Immigration and Refugee Protection Act.

10. This independence is meant to shield refugee decisions from the influence of political and administrative authorities.

11. For example, those making a refugee claim at a Canadian port of entry are required to submit their application in only 15 days (see IRB, 2018).

12. A modified version of the expedited process remains in place today. In the current system, a combination of country of origin and claim type determines if a claim may be diverted to expedited review. For more see IRB (2019).

13. The use of procedural shortcuts and truncations in adjudication of these claims helped optimize productivity. The logic of responsiveness was also valued for increasing output rates.

14. The Lead Cases were successfully challenged and overturned on the basis of creating reasonable apprehension of bias against Roma Hungarians. For more on this see 36 Kozak v. Canada (Minister of Citizenship and Immigration), 2006 FCA 124, A-419-04, A-420-04, para 65. Accessed 22 September 2020 at https://decisions.fca-caf.gc.ca/fcacaf/decisions/en/item/34680/index.do™r=AAAAAQALaW1taWdyYXRpb24B

15. Excessively short timelines also adversely affect claimants’ ability to secure status through applications based on Humanitarian and Compassionate (H&C) grounds. H&C grounds are the last resort for folks who normally do not qualify for permanent residence in Canada, such as failed refugee claimants. The H&C applications are considered in exceptional circumstances and are decided based on determination of claimants’ level of settlement in Canada, their established family ties, and the best interest of any child involved (for more see Government of Canada, 2017), all of which are unlikely to work in the favour of claimants who are rejected and removed before they have time to establish themselves and their families in Canada.

16. For more on accelerated procedures in the UK, Germany, France and Italy (as well as other European countries), see the country reports of the European Council of Refugees and Exiles, available at https://asylumineurope.org/reports/. For a more thorough discussions of the DFT see the report of Detention Action (2011). For more on the American MPP see Human Rights Watch (2021).
